# Comparison of Skeletal Effects of Ovariectomy Versus Chemically Induced Ovarian Failure in Mice

**DOI:** 10.1359/jbmr.080309

**Published:** 2008-03-17

**Authors:** Laura E Wright, Patricia J Christian, Zelieann Rivera, William G Van Alstine, Janet L Funk, Mary L Bouxsein, Patricia B Hoyer

**Affiliations:** 1 Department of Physiology, The University of ArizonaTucson, Arizona, USA; 2 Department of Comparative Pathobiology, Purdue UniversityWest Lafayette, Indiana, USA; 3 Department of Medicine, The University of ArizonaTucson, Arizona, USA; 4 Orthopaedic Biomechanics Laboratory, Department of Orthopedic Surgery, Beth Israel Deaconess Medical CenterBoston, Massachusetts, USA

**Keywords:** menopause, murine, BMD, microarchitecture

## Abstract

Bone loss associated with menopause leads to an increase in skeletal fragility and fracture risk. Relevant animal models can be useful for evaluating the impact of ovarian failure on bone loss. A chemically induced model of menopause in which mice gradually undergo ovarian failure yet retain residual ovarian tissue has been developed using the chemical 4-vinylcyclohexene diepoxide (VCD). This study was designed to compare skeletal effects of VCD-induced ovarian failure to those associated with ovariectomy (OVX). Young (28 day) C57Bl/6Hsd female mice were dosed daily with vehicle or VCD (160 mg/kg/d, IP) for 15 days (*n* = 6–7/group) and monitored by vaginal cytology for ovarian failure. At the mean age of VCD-induced ovarian failure (∼6 wk after onset of dosing), a different group of mice was ovariectomized (OVX, *n* = 8). Spine BMD (SpBMD) was measured by DXA for 3 mo after ovarian failure and OVX. Mice were killed ∼5 mo after ovarian failure or OVX, and bone architecture was evaluated by μCT ex vivo. In OVX mice, SpBMD was lower than controls 1 mo after OVX, whereas in VCD-treated mice, SpBMD was not lower than controls until 2.9 mo after ovarian failure (*p* < 0.05). Both VCD-induced ovarian failure and OVX led to pronounced deterioration of trabecular bone architecture, with slightly greater effects in OVX mice. At the femoral diaphysis, cortical bone area and thickness did not differ between VCD mice and controls but were decreased in OVX compared with both groups (*p* < 0.05). Circulating androstenedione levels were preserved in VCD-treated mice but reduced in OVX mice relative to controls (*p* < 0.001). These findings support that (1) VCD-induced ovarian failure leads to trabecular bone deterioration, (2) bone loss is attenuated by residual ovarian tissue, particularly in diaphyseal cortical bone, and (3) the VCD mouse model can be a relevant model for natural menopause in the study of associated bone disorders.

## INTRODUCTION

Menopause in women occurs when functional ovarian follicles become depleted. At that time, ovarian production of 17β-estradiol, progesterone, and inhibin markedly decreases.(1) After menopause, there is a rapid phase of bone loss and bone microarchitectural deterioration that may last for 5–10 yr, followed by a slower rate of bone loss that continues indefinitely.(2) The classical view of the mechanism underlying bone loss and increased skeletal fragility involves the gradual decline in 17β-estradiol production.(3,4) The cellular basis for these skeletal changes includes enhanced bone turnover, with the balance favoring osteoclast mediated resorption in the absence of estrogen.(4,5) Recent data indicate that, in addition to estrogen, other factors that are altered in the perimenopausal and menopausal periods, such as increased follicle stimulating hormone (FSH) and decreased inhibin, may also directly affect bone metabolism. Changes in gonadal inhibins likely have direct effects on osteoblast and osteoclast activity across the menopausal transition and can be associated with increased bone turnover independent of changes in sex steroids or FSH.(6–8) FSH enhances osteoclast development and survival in vitro, and increased FSH has been reported to be a better predictor of increased bone resorption and decreased BMD than 17β-estradiol.(9–12)

Despite the profound impact of menopause on women's health, animal models that mimic the natural progression through perimenopause and into the postmenopausal stages are currently lacking. Ovariectomy (OVX), or surgical removal of the ovaries, is the most common animal model for studying the mechanisms that underlie the skeletal response to estrogen deficiency. Although widely used, OVX is problematic with regard to reproducing the effects of natural menopause. OVX produces a rapid, dramatic cessation of ovarian function, rather than the gradual decline that occurs in perimenopause. During an early natural menopausal period, inhibin secretion by the ovaries declines, leading to a rise in FSH in the presence of waning 17β-estradiol levels. The combined effect of gradual changes in these hormonal factors on skeletal health cannot be examined in an ovary-deficient animal. Finally, the postmenopausal ovary continues to produce low levels of androstenedione, a hormone known to have bone protective effects, which is not produced in rodents after surgical removal of the ovaries.(13)

A chemically induced mouse model for peri- and postmenopause has been developed using the occupational chemical, 4-vinylcyclohexene diepoxide (VCD).(14–16) VCD selectively destroys ovarian small preantral follicles after repeated daily dosing in mice and rats.(17,18) The mechanism of VCD's highly selective effect on ovarian preantral follicles has been shown in previous studies to be caused by acceleration of the natural process of atresia (apoptosis) through follicle-specific pathways, with no evidence of necrotic changes in ovarian tissue or changes in gene expression that cannot be attributed to ovarian failure.(19–23) Evidence for VCD's lack of toxicity to other tissues or organ systems was published in early work by the National Toxicology Program. In these studies, rats and mice repeatedly exposed to VCD for 2 yr showed that (other than skin lesions at the site of repeated dermal application) the only notable effects were in female ovaries, and no other systems were impacted by this chronic exposure.(24) Because of selectivity of its effects, VCD has been used to cause premature ovarian failure after repeated daily dosing in rodents.(14) After the destruction of preantral follicles, the onset of VCD-induced ovarian failure is associated with fluctuating 17β-estradiol, increasing FSH levels, and irregular estrous cyclicity (i.e., a model for perimenopause).(15,16) Additionally, unlike OVX animals generally used to model menopause, the VCD-treated animal retains residual ovarian tissue (i.e., a model for natural menopause).(14)

Effects of the VCD-induced model of ovarian failure on bone integrity have yet to be compared with the effects of OVX-induced bone loss. We first evaluated the short-term effects of VCD on bone microarchitecture and then compared the skeletal effects of VCD-induced ovarian failure to the effects of OVX. We hypothesized that BMD and bone microarchitecture would be compromised because of ovarian failure in both models, although the timing and magnitude of changes would differ between VCD and OVX animals because of the extended period of impending ovarian failure (VCD) as well as the physiological impact of residual ovarian tissue.

## MATERIALS AND METHODS

### Animals

C57Bl/6Hsd female mice were purchased from Harlan Laboratories, housed in plastic cages, and maintained on 12L/12D cycles at 22 ± 2°C with food (Harlan Teklad 7013 NH-31 modified diet) and water available ad libitum. Animals were allowed to acclimate to the animal facility for 1 wk before initiation of treatment, after which they were randomly assigned to treatment groups. For the first aim, 3-mo-old mice (*n* = 6/group) were dosed daily with VCD (160 mg/kg, IP) or sesame oil (vehicle control, IP) for 15 days and killed immediately after treatment to determine whether VCD has direct short-term effects on bone microarchitecture. For the second aim, 28-day-old animals were dosed daily with VCD or vehicle control for 15 or 17 days (*n* = 6–7/group) and monitored using daily vaginal cytology to determine ovarian failure. Surgical OVX was performed on separate mice at 3 mo of age (*n* = 8), which corresponds to the age at which all VCD-treated mice had undergone ovarian failure. All experiments were approved by the University of Arizona IACUC and conformed to the Guide for the Care and Use of Experimental Animals.

### Vaginal cytology

Estrous cyclicity was determined by daily monitoring of vaginal cytology after dosing with VCD or vehicle. Ovarian failure in VCD-treated mice was assigned when vaginal cytology indicated ≥15 days of persistent metestrus/diestrus or diestrus.(16)

### BMD

In vivo BMD (g/cm^2^) measurements of the lumbar spine (L_2_–L_4_) were made using DXA (PIXImus; GELunar, Madison, WI, USA) with animals under anesthesia (1.25% Avertin).

### Tissue collection

Animals were killed by CO_2_ inhalation at either 3 (for the short-term treatment study) or 8.5 mo of age (for the long-term study). Tibias and femurs from each animal were collected, wrapped in saline-soaked gauze, and frozen at −20°C. The remaining skeleton was fixed in 10% neutral buffered formalin (NBF; 4°C; 48 h) and transferred to 70% ethanol and stored at 4°C for μCT evaluation. Ovaries were collected from VCD-treated and cycling control animals. Uteri, kidneys, adrenals, spleen, liver, lung, heart, brain, intestine, and pituitary tissue from control, OVX, and VCD-treated mice were collected, weighed, and fixed for histopathological evaluation.

### Evaluation of bone microarchitecture by μCT

Femurs and vertebrae were evaluated using a desktop μCT imaging system (μCT40; Scanco Medical AG, Bassersdorf, Switzerland) equipped with a 10-mm focal spot microfocus X-ray tube, as previously described.(25) Transverse CT slices of the distal femoral metaphysis, vertebral body (L_5_), and femoral midshaft were acquired using 12-μm isotropic voxel size. Images were reconstructed, filtered, and thresholded using a specimen-specific threshold, and morphometric parameters were computed using a direct 3D approach that does not rely on any assumptions about the underlying structure.(26–30) For trabecular morphology, the following variables were assessed: bone volume fraction (BV/TV, %), trabecular thickness (TbTh, μm), trabecular separation (TbSp, μm), trabecular number (TbN, 1/mm), connectivity density (ConnD, 1/mm^3^), and structure model index (SMI). For cortical bone, the following variables were assessed: total area (mm^2^), bone area (mm^2^), medullary area (mm^2^), cortical bone volume fraction (BA/TA, %), and cortical thickness (CortThick, μm).

### Hormone assays

Plasma 17β-estradiol and androstenedione were determined by Coat-A-Count RIAs 3 wk and 2 mo after ovarian failure/OVX, respectively (Diagnostic Products, Los Angeles, CA, USA).

### Data analysis

Differences between groups and over time within groups were analyzed by one-way ANOVA. Posthoc tests (Fisher's protected least significant difference) were used where appropriate. All analyses were performed using Instat software (Graphpad, San Diego, CA, USA). Differences were considered significant at *p* < 0.05.

## RESULTS

### Short-term effects of VCD on bone

Two-weeks of VCD treatment had no direct effect on bone morphology, because bone microarchitecture in the vertebrae, distal femur, and femoral midshaft was not different between VCD and vehicle-treated mice killed immediately after VCD dosing (before ovarian failure; data not shown). Thus, these two groups were combined to form a young (3 mo old) control group (*n* = 12).

### Ovarian failure

Immature (28 day old) mice dosed daily with VCD exhibited ovarian failure at a mean of 48.1 ± 2.7 days after the onset of dosing, with a range of 38–64 days. Vehicle-treated control animals exhibited regular estrous cycles throughout the course of the experiment (4.7 ± 0.3 days/cycle).

### BMD

**FIG. 1:**
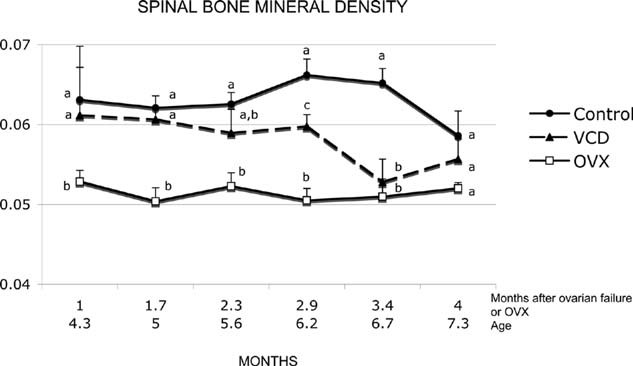
Temporal changes in SpBMD in VCD-treated vs. OVX mice assessed by DXA (values = mean ± SE, *n* = 5–10/group). Female C57Bl/6Hsd mice were dosed daily with vehicle control or VCD (16.0 mg/kg/d). A subset of control animals underwent OVX corresponding to the time of VCD-induced ovarian failure. Different letters (a, b, or c) represent different (*p* < 0.05) mean values.

Lumbar spine BMD (SpBMD) was monitored by DXA at time points between 1 and 4 mo after ovarian failure for VCD-treated animals or after OVX for OVX animals (Fig. 1). Relative to vehicle-treated controls, SpBMD was significantly lower in OVX mice as early as 1 mo after surgery (*p* < 0.05). SpBMD remained significantly lower than control animals throughout the course of the experiment until 4 mo after OVX (corresponding to 7 mo of age), at which time vehicle-treated controls began to experience a decline in bone mass. In contrast to this early and persistent decrease in SpBMD in OVX animals, VCD-treated animals experienced a slower decline in SpBMD relative to vehicle-treated control animals, and SpBMD was only different from controls at 3 mo after ovarian failure (*p* < 0.05). At 4 mo after ovarian failure or OVX, SpBMD was not different between the groups.

### Bone microarchitecture

**FIG. 2:**
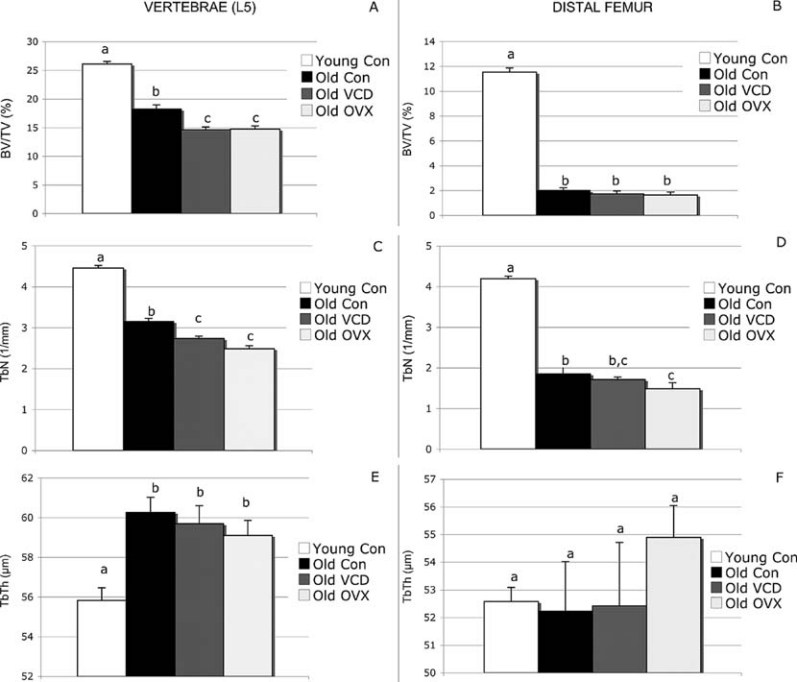
Trabecular bone architecture at the fifth lumbar vertebral body and distal femoral metaphysis in old (age 8.5 mo) and young (age 3 mo) female C57Bl/6Hsd mice assessed by μCT (values = mean ± SE, *n* = 6–12/group). Animals were dosed daily with vehicle control or VCD (160 mg/kg/d). Old VCD and OVX animals were 5.3 mo postovarian failure or ovariectomy. The following variables were assessed for trabecular morphology: bone volume fraction (BV/TV, %), trabecular thickness (TbTh, μm), trabecular separation (TbSp, μm), trabecular number (TbN, 1/mm), connectivity density (ConnD, 1/mm^3^), and structure model index (SMI). Different letters (a, b, or c) represent different (*p* < 0.05) mean values.

Approximately 5.3 mo after ovarian failure/OVX (i.e., 8.5 mo of age), vertebral trabecular BV/TV, TbN, and ConnD were decreased (*p* < 0.01), and TbSp was increased (*p* < 0.01) in both VCD-treated and OVX mice relative to vehicle-treated age-matched controls (Table 1; Fig. 2). In the distal femur, TbN was decreased (*p* < 0.01) and TbSp increased (*p* < 0.001) in OVX relative to controls, whereas no change was detected in the VCD-treated group relative to vehicle-treated controls. Moreover, in the OVX group, TbSp was increased (*p* < 0.05) relative to VCD-treated animals in both trabecular compartments.

**Table 1:** Bone Microarchitecture at the Lumbar Spine, Distal Femur, and Femoral Midshaft, Assessed by μCT

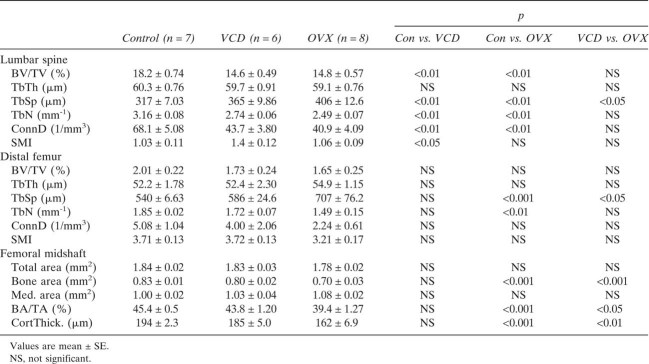

**FIG. 3:**
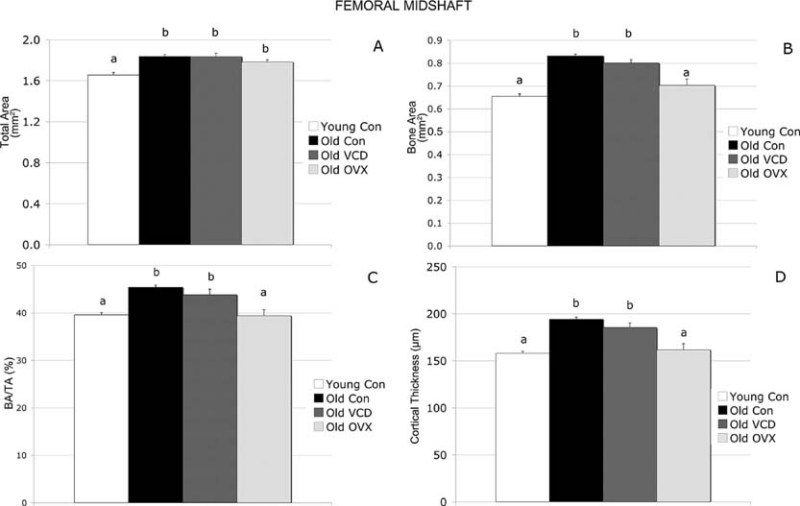
Midfemoral cortical bone in old (age 8.5 mo) and young (age 3 mo) in female C57Bl/6Hsd mice, assessed by μCT (values = mean ± SE, *n* = 6–12/group). Animals were dosed daily with vehicle control or VCD (160 mg/kg/d). Old VCD and OVX animals were 5.3 mo postovarian failure or ovariectomy. For cortical bone, the following variables were assessed: total area (mm^2^), bone area (mm^2^), cortical bone volume fraction (BA/TA, %), and cortical thickness (CortThick, μm). Different letters (a, b, or c) represent different (*p* < 0.05) mean values.

No changes were detected at the femoral midshaft in VCD-treated animals relative to vehicle-treated age-matched controls (Table 1; Fig. 3). In contrast, in OVX animals, cortical bone area, bone area fraction, and thickness were reduced relative to VCD-treated and vehicle-treated controls (*p* < 0.05 for all).

To assess underlying age-related changes, we compared the young (3 mo old) control group to the vehicle-treated group killed at ∼8.5 mo of age. Aging was associated with significant trabecular bone deterioration. However, compared with young controls, midfemoral size and cortical bone area and thickness were higher in the older VCD- and vehicle-treated mice but not OVX mice (Table 2; Fig. 3).

**Table 2:** Bone Microarchitecture at the Lumbar Spine, Distal Femur, and Femoral Midshaft in Young (3 mo) vs. Old (8.5 mo) Mice, Assessed by μCT

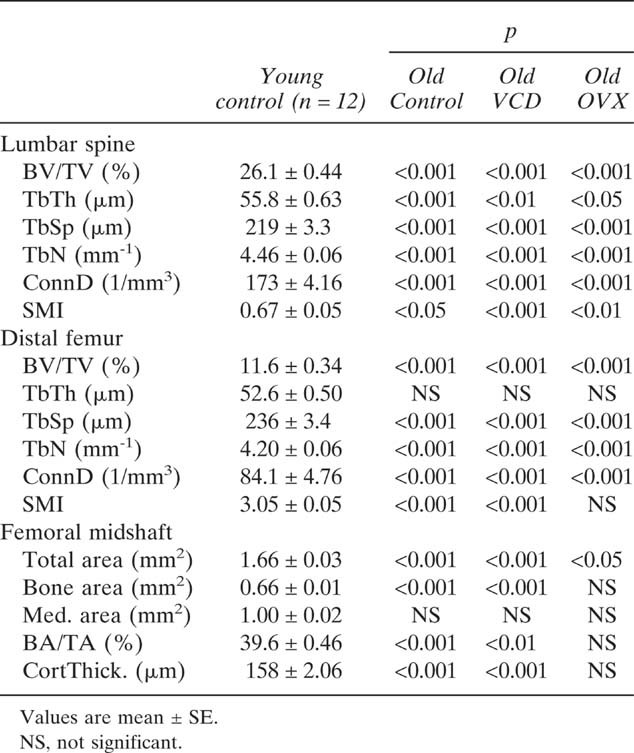

### Circulating androstenedione levels

**FIG. 4:**
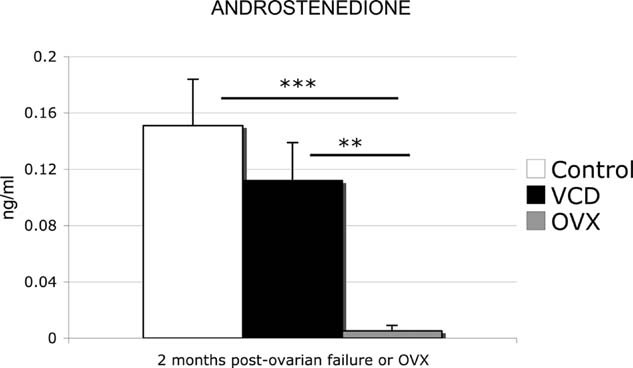
Effect of VCD-induced ovarian failure or OVX on circulating androstenedione levels in female C57Bl/6Hsd mice 2 mo after ovarian failure or ovariectomy (values = mean ± SE, *n* = 5/group). Animals were dosed daily with vehicle control or VCD (160 mg/kg/d). Circulating levels of androstenedione were measured by RIA (**p < 0.01; ***p < 0.001).

Circulating androstenedione levels were not different between control and VCD-treated animals 2 mo after ovarian failure (Fig. 4). In contrast, androstenedione levels in OVX animals 2 mo after OVX were significantly lower than both control (−97%, *p* < 0.001) and VCD animals (−95%, *p* < 0.01).

### Circulating 17β-estradiol levels

**FIG. 5:**
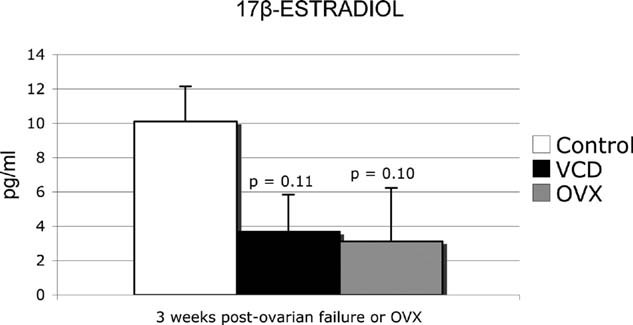
Effect of VCD-induced ovarian failure or OVX on circulating 17β-estradiol levels in female C57Bl/6Hsd mice 3 wk after ovarian failure or OVX (values = mean ± SE, *n* = 5/group). Animals were dosed daily with vehicle control or VCD (160 mg/kg/d). Circulating levels of 17β-estradiol were measured by RIA.

Circulating levels of 17β-estradiol in VCD-treated and OVX mice were reduced by >50% compared with cycling controls 3 wk after ovarian failure or OVX (Fig. 5). Terminal (8.5 mo of age) 17β-estradiol levels were undetectable in both VCD and OVX groups (data not shown).

### Tissue weights

The effect of VCD dosing and OVX on tissue weights was determined at the time of skeletal collection and expressed as percent of total body weight (Table 3). Consistent with the known hyperphagia associated with estrogen deficiency, both OVX and VCD-treated animals had increased body weight relative to controls, although this reached statistical significance only in the VCD-treated mice (*p* < 0.05). There was no difference between tissue weight of adrenals, spleen, kidneys, or liver between vehicle-treated controls, OVX, and VCD-treated animals. Ovarian weights were decreased in VCD-treated animals relative to controls (*p* < 0.0001), and uterine weights were decreased in both OVX and VCD-treated groups relative to controls (*p* < 0.001 and *p* < 0.01, respectively). Uterine weights in OVX animals were also lower (*p* < 0.05) than in VCD-treated animals. Histopathological evaluation of OVX and VCD-treated animals confirmed morphological evidence of uterine atrophy. Importantly, there were no pathological effects observed microscopically in uteri, kidneys, adrenals, spleen, liver, lung, heart, brain, intestine, or pituitary tissues from VCD-treated mice.

**Table 3:** Tissue Weights Expressed as Percent of Total Body Weight

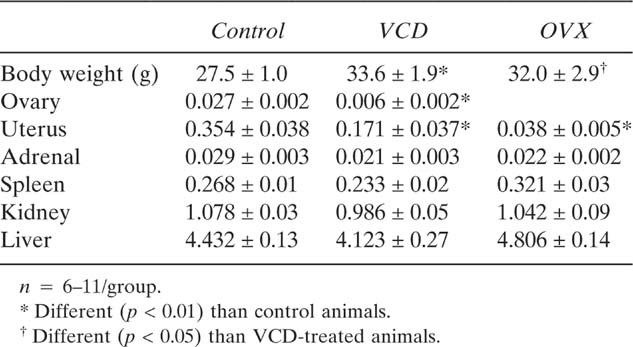

## DISCUSSION

The overall purpose of this study was to compare skeletal effects of ovarian failure in a chemically induced mouse model of menopause (VCD treatment) with those seen in ovariectomized (OVX) mice. The first aim was to evaluate short-term effects of VCD treatment on trabecular or cortical bone microarchitecture. μCT measurements in 3-mo-old mice killed immediately after the completion of 15 days of VCD dosing showed that there were no direct effects of VCD treatment on bone microarchitecture. Additionally, based on assessment of tissue weights and histopathological evaluation at necropsy, there were no effects of VCD or OVX on tissues other than the ovary and uterus or the uterus, respectively. The loss of ovarian weight in VCD-treated animals can be attributed to ovarian failure, whereas the loss of uterine weight in VCD-treated and OVX animals can be attributed to loss of uterotropic effects of 17β-estradiol.(15) Thus, the bone-related effects seen in this study in VCD-treated animals are most likely the result of loss of ovarian function rather than any direct toxic effects of VCD on bone.

C57Bl/6Hsd mice dosed with VCD underwent ovarian failure on an average of 48 days after the onset of dosing (as defined by 15-day persistent diestrus). As expected, both OVX and VCD-treated animals had reduced 17β-estradiol 3 wk after either surgery or chemical induced ovarian failure. Loss of estrogen's bone protective effects leads to a decline in bone mass in women after menopause.(31,32) OVX animals experienced a significant loss in SpBMD at 1 mo after ovariectomy, whereas SpBMD in VCD-treated animals was not reduced to that level for 2.5 more months. Thus, despite ovarian failure and subsequently low levels of circulating 17β-estradiol, VCD-treated animals did not experience as rapid a decline in SpBMD as seen in OVX animals.

Whereas both VCD-induced ovarian failure and OVX led to a significant deterioration of trabecular bone microarchitecture, there was a trend for greater and more consistent declines in OVX versus VCD-treated mice. Furthermore, cortical bone at the femoral midshaft was preserved in VCD-treated mice, whereas OVX mice showed significant cortical bone loss compared with age-matched controls.

Androstenedione may provide insight into the slower and less dramatic loss of bone integrity in the VCD model for natural menopause. Two months after ovarian failure, circulating androstenedione levels remained normal and significantly higher in VCD-treated animals relative to OVX animals. As the mammalian ovary loses follicles by ovulation or atresia, theca interna cells transition into interstitial cells responsible for the production of androgens.(33) Previous studies have shown that residual ovarian tissue in the follicle-deplete, VCD-treated mouse produces androstenedione,(14) a hormone known to preserve bone mass in the rodent skeleton by restraining effects on osteoblastogenesis and osteoclastogenesis.(34,35) Thus, continued production of androgens after ovarian failure in the VCD-treated group is likely responsible for the more gradual decline in SpBMD over time, a hormone profile similar to that seen over the course of the natural human peri- and postmenopausal transition. It has recently been confirmed that the human postmenopausal ovary produces androgens well after menopause.(36) Therefore, in this regard, the VCD-treated mouse more appropriately mimics natural menopause than the OVX model.

At 4 mo after ovarian failure/OVX for treatment groups, animals were ∼6 mo of age, and SpBMD began to decline in control animals. Relative to young mice, the aged vehicle-treated control animals had also experienced a significant loss in vertebral and femoral bone microarchitecture over the course of the study, despite normal estrous cyclicity. Recent studies have shown that female C57Bl/6J mice experience a steady decline in trabecular bone beginning at 2–3 mo of age and that deterioration in trabecular bone architecture is more severe in the metaphyseal region of long bones than in the vertebral body.(25,37) Thus, failure to observe drastic differences between OVX and VCD treatment in older animals in this study, particularly in the distal femur, was likely the result of ongoing age-related skeletal deterioration that muted the effects of VCD treatment or OVX.

Increased circulating FSH levels are seen during peri- and postmenopause in women because of loss of negative feedback on the anterior pituitary by ovarian steroids and inhibin.(1) Increasing FSH, therefore, is used as an early predictor that precedes the onset of menopause. It has been hypothesized that increased loss of bone mass seen in menopausal women is because of direct effects of FSH on bone.(9,38) The proposed mechanism is through FSH stimulation of TNFα production by bone marrow granulocytes and macrophages. This increase in TNFα expands the number of bone marrow osteoclast precursors resulting in hypogonadal bone loss. In the VCD-treated murine model of menopause, we have previously shown that circulating FSH levels increase before ovarian failure after the onset of dosing in mice.(14,15) In this study, SpBMD loss was not detected until 2.9 mo after ovarian failure, which corresponds to day 154 after the onset of dosing. Therefore, these findings suggest that exposure of murine bone to 4 mo of increased circulating FSH does not directly cause loss of bone integrity. BMD measurements by DXA, however, may not be sufficiently sensitive to detect early changes in trabecular bone microarchitecture. Biochemical markers of bone turnover may be useful in this regard.

One shortcoming of this study is that direct, short-term effects of VCD on bone microarchitecture was evaluated in animals that were 3 mo of age, whereas ovarian failure was induced with VCD in mice that were 28 days old at the start of dosing. It is possible that the skeleton of young, growing animals may respond differently to VCD than the skeleton of mature, 3-mo-old animals. Additionally, the fact that we waited several months after ovarian failure to assess bone microarchitecture meant that mice had undergone significant age-related bone loss by that time, as clearly seen in comparisons between the young and old control animals. Despite this complication, we were still able to detect significant differences in bone microarchitecture in VCD-treated and OVX animals that can likely be attributed to loss of ovarian function because control animals were still cycling.

To confirm that VCD's negative impact on bone could be attributed exclusively to ovarian failure, our laboratory previously conducted a study in which the effects of VCD treatment and VCD treatment + subcutaneous E_2_ pellets on BMD and bone microarchitecture were evaluated in mice.(39) Similar to the findings in this study, VCD-treated mice had significantly lower femoral BMD, lower vertebral trabecular BV/TV and number, and increased trabecular separation relative to vehicle-treated mice. Consistent with previous reports of anabolic effects of estrogen in mice,(40–45) VCD + E had increased femoral BMD, and markedly improved trabecular bone parameters at the vertebrae and distal femur compared with both VCD- and vehicle-treated animals. These initial findings showed that VCD-induced ovarian failure caused bone deterioration, and effects of VCD treatment on bone could be reversed with exogenous estrogen administration. Furthermore, it confirmed the need for a direct comparison of the skeletal effects of VCD-induced ovarian failure to that of OVX, as reported in this study.

In summary, our results showed that VCD treatment does not have direct effects on bone, and that, as a result of chemically induced ovarian failure, bone mass and microarchitecture are compromised in the VCD-treated murine model for peri- and postmenopause. Compared with the OVX model for menopause, skeletal changes in the VCD model appear more slowly and are of lower magnitude. These differences are most likely caused by the more physiological approximation of natural menopause with gradual onset of ovarian failure (model for perimenopause) and retention of androgen-producing residual ovarian tissue (model for postmenopause) after ovarian failure in VCD-treated animals. These findings indicate that the VCD model may be quite useful for future studies related to menopause-associated bone disease, including both the perimenopausal and postmenopausal periods. Although the OVX model is limited in relevance for natural peri- and postmenopause, it is useful for the assessment of potential treatments for osteopenia in hypogonadal animals caused by the resultant accelerated bone loss that can be achieved in a relatively short amount of time. Thus, both surgical (OVX) and natural (VCD) menopause models bring unique strengths to the study of estrogen deficiency on skeletal integrity, and future studies using these combined models might be useful for producing a complementary approach.
